# Computerized Decision Aids for Shared Decision Making in Serious Illness: Systematic Review

**DOI:** 10.2196/medinform.6405

**Published:** 2017-10-06

**Authors:** Anna Staszewska, Pearl Zaki, Joon Lee

**Affiliations:** ^1^ Health Data Science Lab School of Public Health and Health Systems University of Waterloo Waterloo, ON Canada

**Keywords:** decision making, decision aids, evidence-based medicine, user-computer interface, chronic disease

## Abstract

**Background:**

Shared decision making (SDM) is important in achieving patient-centered care. SDM tools such as decision aids are intended to inform the patient. When used to assist in decision making between treatments, decision aids have been shown to reduce decisional conflict, increase ease of decision making, and increase modification of previous decisions.

**Objective:**

The purpose of this systematic review is to assess the impact of computerized decision aids on patient-centered outcomes related to SDM for seriously ill patients.

**Methods:**

PubMed and Scopus databases were searched to identify randomized controlled trials (RCTs) that assessed the impact of computerized decision aids on patient-centered outcomes and SDM in serious illness. Six RCTs were identified and data were extracted on study population, design, and results. Risk of bias was assessed by a modified Cochrane Risk of Bias Tool for Quality Assessment of Randomized Controlled Trials.

**Results:**

Six RCTs tested decision tools in varying serious illnesses. Three studies compared different computerized decision aids against each other and a control. All but one study demonstrated improvement in at least one patient-centered outcome. Computerized decision tools may reduce unnecessary treatment in patients with low disease severity in comparison with informational pamphlets. Additionally, electronic health record (EHR) portals may provide the opportunity to manage care from the home for individuals affected by illness. The quality of decision aids is of great importance. Furthermore, satisfaction with the use of tools is associated with increased patient satisfaction and reduced decisional conflict. Finally, patients may benefit from computerized decision tools without the need for increased physician involvement.

**Conclusions:**

Most computerized decision aids improved at least one patient-centered outcome. All RCTs identified were at a High Risk of Bias or Unclear Risk of Bias. Effort should be made to improve the quality of RCTs testing SDM aids in serious illness.

## Introduction

### Background and Significance

Shared decision making (SDM) is important in achieving patient-centered care, as it involves both the patient and the health care provider in medical decision making [1]. More than one reasonable treatment decision exists for the majority of medical decisions, and thus, patient involvement is of great value [2]. As patient involvement in treatment decisions increases, it is more likely that the treatment decision will be consistent with their preferences, lifestyles, and goals [3]. Competing values and perspectives between physicians and patients are often compounded by ineffective patient-provider communication regarding disease and goals of treatment [4]. Patients may choose treatment options based on erroneous outcome expectations and misunderstanding of the disease. For example, in a study by Weeks et al [5], 69.0% (490/710) of patients with metastatic lung cancer and 80.9% (391/483) of patients with metastatic colorectal cancer did not understand that chemotherapy was not likely to be curative.

SDM tools such as decision aids are intended to inform the patients with regard to the risks, benefits, and trade-offs associated with a decision [6]. When used to assist in decision making between treatments, decision aids have been shown to reduce decisional conflict, increase ease of decision making, and increase modification of previous decisions [7,8]. Furthermore, it may be that patients who are informed about their disease because of the use of decision aids are less likely to choose nonbeneficial treatment [7].

Computerized decision aids can offer personalized evidence-based care, and if they are presented in an SDM capacity they can result in treatment decisions that respect the autonomy and preferences of the patient. Additionally, technological advances that use and process electronic health record (EHR) data may allow for the development of large-scale, low-cost assessments that can improve patient goals [9]. Computerized decision aids may provide additional benefits over traditional paper or video tools, as they have the potential for individualized content, a greater degree of interaction, and scalability [10].

A recent systematic review by Austin et al [11] synthesized the evidence for the use of decision aids in serious illness through the evaluation of randomized controlled trials (RCTs) and non-RCTs. However, the review by Austin et al [11], a relevant Cochrane systematic review on SDM tools by Stacey et al [8], and a review on the use of video decision aids in advanced care planning [12] do not focus on the ability of *computerized* decision tools to improve patient-centered outcomes. A focus on computerized decision aids is both timely and necessary because of the possibility of greater personalization of computerized decision aids, which is congruent with the goal toward individualized treatment plans. Additionally, computerized decision aids offer greater scalability over the traditional static decision aids [10]. Finally, a systemic move toward the digitization of health data allows for the natural progression of its use in decision support systems. Few other systematic reviews have focused on computerized decisions tools. Syrowatka et al [13] conducted a systematic review and meta-analyses to classify the features that have been integrated into computerized decision aids and assessed whether these features enable higher-quality decision making. Sheehan and Sherman [14] evaluated the effectiveness of various computerized decision aids in preference-sensitive health-related contexts such as treatments, screening, genetic testing, and risk-management decisions. Their study found that computerized decision aids were efficacious in improving decision-specific knowledge, reducing decisional conflict, and facilitating satisfaction with the decision-making process. Murray et al [15] examined the use of interaction health communication applications (IHCAs), a specific format of a computerized decision aid, for people with chronic disease. The findings have suggested that IHCAs are able to increase patients’ knowledge and sense of support as well as improve clinical outcomes. These studies provide a foundation upon which to further assess computerized decision aids. Missing from the current literature is a review of the available computerized decision aids that specifically address shared decision making by seriously ill patients.

### Objective

This systematic review builds on the work of Austin et al [11] and assesses the impact of computerized decision aids on patient-centered outcomes of seriously ill patients. Austin et al [11] defined serious illness to include “critical life-threatening illness, advance stages of major chronic diseases or multi-morbidity and frailty.” The tools reviewed by Austin et al [11] included print, video, or Web-based formats. For the scope of this review, serious illness will refer to critical, life-threatening illness, chronic disease, multimorbidities, and frailty. This definition of serious illness is a modified version of the definition put forth by Austin et al [11]; the scope of the definition has been broadened to include all stages of chronic disease. Chronic disease is a growing burden and the most common and costly of all health problems; 86% of all health care spending in the United States in 2010 was for individuals with one or more chronic medical conditions [16]. Additionally, chronic diseases are generally long term, progressive in severity, rarely curable [17,18], and thus, may require many decisions to be made over a lifetime.

## Methods

The preferred reporting items for systematic reviews and meta-analyses (PRISMA) checklist for systematic reviews was followed for this review. The study was not registered with the International Prospective Register of Systematic Reviews (PROSPERO), and therefore, registration information is not included.

### Information Sources

PubMed and Scopus databases were searched in February 2016. The search was conducted without a limitation on the year of publication. The search strategy terms were based on the terms used in the systematic review of similar topic by Austin et al [11] and modified based on the specific technological interests of this paper.

The search terms utilized in the PubMed database were as follows:

(Computer*[tw] OR electronic health records [MESH] OR internet[MESH] OR electronic medical record*[tw] or website[tw] or web site[tw]) AND (decision making[tw] OR decision support[tw] OR decision support techniques[MESH]) AND (shared[tw] OR patient[MESH] OR patient*[tw] OR patient*centered OR family[tw] OR physician patient relations[MESH] OR surrogate[tw] OR professional family relations[MESH] OR professional family relations[MESH]) AND (terminal[tw] OR chronic[tw] OR advanced[tw] OR severity[tw] OR severe[tw] OR failure*[tw] OR end stage[tw] OR endstage[tw] OR dying[tw] OR Intensive Care Units[MeSH] OR intensive care[tw] OR ICU[tw] OR hospice*[tw])

The above search was then modified for the Scopus database:

(TITLE-ABS-KEY (“shared decision making” OR sdm OR “patient preferences”) AND TITLE-ABS-KEY (illness OR disease OR “intensive care” OR serious) AND TITLE-ABS-KEY (web OR “web based” OR internet OR “computerized decision support” OR cdss OR “decision support” OR technology OR “electronic health record” OR “electronic medical record” OR ehr))

### Study Selection

Papers extracted from the search results mentioned SDM tools/aids, communication tools/aids, or SDM in relation to an illness or disease in the title or abstract. The abstracts and/or full text were then reviewed; papers were included if the study design was determined to be an RCT. Papers that assessed the use of noncomputerized tools or aids such as videos or pamphlets were excluded as the purpose of this review was to consider computerized decision tools. Tool formats included were Web-based, EHR portals, or computerized decision support software. Included RCTs had to discuss the use of computerized decision aids in serious illness as defined in the introduction. Finally, the paper had to discuss the tool in relation to aspects of SDM such as reducing decisional conflict and increasing knowledge. Tools were included if they were for the use of patients and/or family of patients. The patient population considered included both adults and children living with serious illness.

The references in the selected papers were hand-searched for relevant papers. Data from the final papers were manually extracted. Only published papers and papers in English were included in the study. The selection of papers was completed by one investigator.

### Quality Assessment

Papers were graded on quality using a Cochrane Risk of Bias Tool (Modified) for Quality Assessment of Randomized Controlled Trials. The quality assessment included the following study validity domains: selection bias, performance bias, detection bias, attrition bias, reporting bias, and other bias. Studies were assessed as either High, Low, or Unclear Risk of Bias. Identified problems in one domain would result in the study being labeled as “High Risk of Bias.” Assessment of the quality of the selected papers was completed by 2 investigators. In case of a disagreement between the 2 investigators completing quality assessment, a third investigator was consulted.

### Analysis

Study characteristics of all included RCTs were described according to PRISMA systematic review guidelines. All patient-centered outcomes in relation to SDM or communication were described, regardless of whether they differed significantly from the control. Patient-centered outcomes extracted from studies varied and included satisfaction with decision, decisional conflict, clinical outcomes, knowledge, preparation for decision making, emotional well-being, perceived involvement in medical decision making, patient expectations, satisfaction with physician discussion, parental activation, and number of school or work days missed. As *P* values for study outcomes were available for the majority of patient-centered outcomes measured in the RCTs, they were used to describe the efficacy of the interventions.

## Results

**Figure 1:**
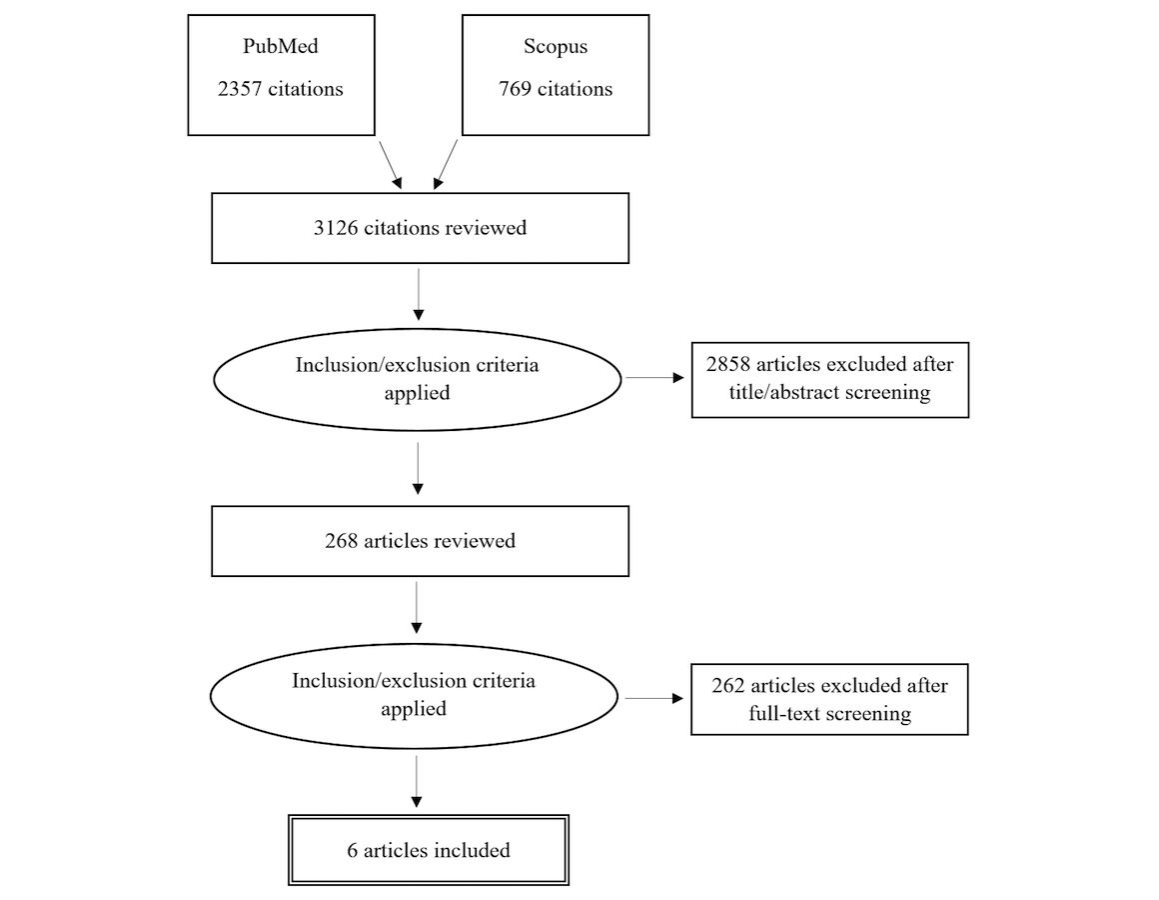
Literature search and selection.

A total of six papers describing RCTs of SDM tools for serious illness were selected and reviewed (Figure 1): three papers described the efficacy of Web-based tools [19-21]; one paper described a tool that operated through an EHR portal [22]; and two papers described interactive computer application tools [23,24]. The ensuing sections will describe each paper in more depth. The effects of the tools on patient-centered outcomes are also shown in Multimedia Appendix 1 [19-24].

The results suggest that computerized decision aids may be used for various types of serious illnesses in a variety of different health care settings to assist both patients and clinicians in decision making. Generally, the selected RCTs demonstrated that computerized decision aids were able to reduce decisional conflict [19,21,23], improve satisfaction with decisions [19,23], and improve health outcomes [18,19,24]. Other factors that may have influenced the use and efficacy of the computerized aids included type and severity of illness [19,21,22,24], patients’ age [23,24], and patients’ education and computer literacy [23,24].

Each of the formats of the computerized decision aids included common features among them. The Web-based decision tools commonly used surveys or questionnaires to ascertain patient preferences, which were then used to guide patient-physician communication or to provide treatment options. The EHR portal decision tool featured the ability for patients to track relevant information and provided educational content, both of which were ultimately used to guide treatment plans. Estimates of treatment efficacy and prognosis were common in interactive computer applications. Of the six selected RCTs, Web-based decision tools were described by Meropol et al [19] for metastatic cancers, van der Krieke et al [20] for nonaffected psychosis, and Weymann et al [21] for type 2 diabetes (T2D) and chronic lower back pain (CLBP). An EHR portal decision tool was used for the management of asthma in the RCT by Fiks et al [22]. Hochlehnert et al [23] and Peele et al [24] described the use of interactive computer applications for the treatment and management of fibromyalgia and breast cancer, respectively.

### Web-Based Decision Tools

In a single-blind RCT, Meropol et al [19] tested an interactive Web-based communication aid (CONNECT) for patients with solid metastatic tumors. Cancer patients were randomized into (1) control group, (2) CONNECT aid with communication skills training (CST) and summary report to the physician, and (3) CONNECT aid and CST without physician summary report. The control group was directed to the National Cancer Institute’s website and received usual care.

There were no statistically significant differences between the two different intervention arms on any of the satisfaction or decisional conflict responses; the summary report for the physician did not improve outcomes. Intervention arms were combined and analyzed against the control arm. Participants assigned to intervention groups had higher levels of satisfaction with discussions about the format of physician communications and quality of life issues but did not differ in satisfaction of discussion regarding diagnosis/prognosis, treatment options, or support community services. Those in the intervention arms found that CONNECT made it easier to reach treatment decisions and were more satisfied with their treatment choice. Participants in the intervention groups had decreased expectations of severe side effects with standard or experimental therapy. The CONNECT intervention was associated with increased satisfaction with overall communication in those with postsecondary education. Additionally, patients in the intervention arm reporting a lower baseline quality of life had greater satisfaction with overall communication.

The study was limited by a racially and ethnically homogenous sample population that was mostly gathered from large cancer centers. Furthermore, the eligibility criteria limited the study to include only those with personal Internet access or those who could arrive early to their appointments to access computers on-site. Additionally, patients in the control groups were directed to the National Cancer Institute website, where extensive searching by the patients may result in a reduced difference between groups. Furthermore, the merging of the intervention groups may place the study at risk of reporting bias. Using the modified Cochrane Risk of Bias Tool for Quality Assessment of Randomized Controlled Trials, the RCT by Meropol et al [19] was rated at High Risk of Bias.

An RCT by van der Krieke et al [20] examined the capability of a Web-based intervention to facilitate SDM for people with psychotic disorders. Patients in the intervention group were given usual care and access to a Web-based tool to support SDM. The control group was given usual care.

Perceived involvement in medical decision making did not differ from patients in the control condition. There were no differences in self-reported satisfaction with care between study arms. However, within the intervention group, those who received the allocated intervention reported lower satisfaction with care in comparison with those who did not receive the intervention.

The study demonstrated a low response rate (29.2%, 73/250) and a moderate participation rate. Furthermore, the study protocol was weakly implemented; not all participants in the intervention group were offered the possibility to use the decision aid, and treatment evaluation meetings where the SDM process would have been used to guide treatment plans did not always occur. The authors do not provide sufficient information regarding the blinding process, if any, that was implemented in the study; therefore, there is unclear risk of selection, performance, and detection bias. The study by van der Krieke et al [20] was rated at Unclear Risk of Bias.

A Web-based, tailored, interactive health communication application for patients with T2D or CLBP was tested in an RCT by Weymann et al [21]. The intervention group received the Web-based tailored communication tool that provided basic information on T2D and CLBP, along with treatment options in an interactive dialogue format. The control group received an untailored Web-based communication tool that was not presented in a dialogue format.

Intention-to-treat analysis, which used the baseline data, found no statistically significant differences between the groups; however, there was a significant difference between T2D and CLBP users, indicating higher knowledge scores in the T2D group. Conversely, sensitivity analysis, which used data from the available cases, found that participants using the tailored system displayed more knowledge immediately after the first visit than those in the control group. Additionally, those in the intervention group had more emotional well-being as identified by a subscale of a patient empowerment scale at the 3-month follow-up. Sensitivity analysis did not result in significant differences between the intervention and control groups in decisional conflict and preparation for decision making.

The sample population was only limited to those with personal Internet access, which may not be representative of the general population. Additionally, the study did not assess outcome criteria at baseline or address potential confounders, both of which make it unclear whether any observable differences were a result of the intervention or other factors. The measure used to assess T2D/CLBP knowledge, a primary outcome of the trial, was also not validated. Moreover, despite blinding of the participants, the use of the dialogue format may have allowed participants to identify the intervention. The study by Weymann et al [21] is therefore at a risk of detection bias and was rated at High Risk of Bias.

### EHR Portal Decision Tool

An RCT by Fiks et al [22] tested the impact of an EHR-linked patient portal with decision support directed at both families and clinicians on asthma outcomes in pediatric patients. The intervention consisted of an EHR-based Web portal, MyAsthma, which provided decision support to both families and clinicians. The families in the control arm did not have access to the portal, but their physicians had access to a clinician-focused decision support system.

The authors reported no statistically significant differences between the control and intervention groups’ satisfaction with asthma care or medication receipt, but data were not made available in the study report. There was no effect on parental knowledge, skills, and confidence. Parents in the intervention group had a significant decrease in the number of days of work missed in comparison with the controls. Analysis indicated an improvement of the frequency of asthma flares in the intervention group compared with the control group. There were no differences in quality of life measurements between the two groups; however, compared with the control group, families of intervention group reported fewer emergency department visits and hospitalization over 6 months. Portal use was also found to be greater in parents of children with moderate to severe asthma than those whose children had mild persistent asthma.

As the participants were recruited based on referrals by physicians or EHR rosters, the sample is considered a convenience sample and its representativeness is unclear. Also, because of the small sample size, randomization did not result in a balance between intervention and control groups in terms of asthma severity. The inadequate randomization of participants places the study at a risk of selection bias, and therefore, the study by Fiks et al [22] was rated at High Risk of Bias.

### Interactive Computer Applications

An RCT by Hochlehnert et al [23] examined the impact of a computerized information tool with and without physician communication training on SDM in patients with fibromyalgia. Patients were randomized into two study arms: (1) a shared decision group (SDM group) that was given a computer-based information tool and then an opportunity for consultation with a physician with communications training and (2) an information-only group (Info group) that was also given the computer-based information tool but was treated by doctors without communications training with no opportunity for feedback and discussion after viewing the tool.

There was no significant difference in satisfaction with decision or decisional conflict, as well as assessment of information tool between the two groups. The two groups were merged for analysis, and it was found that those who were satisfied with the information presented in the tool experienced more satisfaction with their decision and experienced less decisional conflict. Furthermore, those who perceived the tool to be useful in a general practitioner’s office and were satisfied with introduction of the tool (ie, training) were more likely to be satisfied with their decision.

The authors do not provide sufficient information regarding the blinding process that was implemented in this trial; therefore, the risk of performance and detection bias is unclear. The study by Hochlehnert et al [23] was rated at an Unclear Risk of Bias.

An RCT by Peele et al [24] compared rates of breast cancer adjuvant therapy between an intervention group that received a patient-specific decision aid in the form of a computer program and a control group that received an informational pamphlet. Women with breast cancer, who completed their primary surgical treatment, were candidates for adjuvant therapy (chemotherapy, hormonal, or combination therapy) and were randomized into control or intervention groups. The computer program, Adjuvant!, produced prognostic estimates of survival with and without adjuvant therapy by using estimates of individual patient prognosis as well as estimates of the efficacy of adjuvant therapy options.

Women who received the decision aid were significantly less likely to choose adjuvant therapy than those in the control group; one-third fewer women in the intervention group received adjuvant therapy than their counterparts in the control group. The impact of the decision aid based on tumor severity found that the participants in the intervention group with low tumor severity rejected adjuvant therapy significantly more often than the participants in the control group. Generally, women with higher tumor severity, younger women, and women with a university-based physician were more likely to choose adjuvant therapy.

Neither patients nor clinicians were blinded in this study, indicating risk for performance and detection bias. A higher proportion of university-based physicians were randomized into the intervention group, which places the study at a risk of selection bias as well. The study by Peele et al [24] was therefore categorized as at a High Risk of Bias.

## Discussion

### Principal Findings

Study results from the six RCTs discussed in the Results section demonstrate that computerized decision aids have the potential to improve patient-centered outcomes. Furthermore, decision aids have differing impacts on various patient-centered outcomes that can possibly be attributed to tool design, user characteristics, or type of disease. Coincidentally, in this review, each of the selected RCTs employed computerized decision aids in management of chronic illnesses, although this was not specified in the search strategy. Furthermore, the small number of studies that are included in this review also suggests that there is still much work to be done in this area. Of the six computerized decision aids discussed in this review, only the tool used by Hochlehnert et al [23] is available online in German (accessed here: www.fibronet.org).

Decisional conflict was addressed in four RCTs [19-21,23]. The CONNECT decision aid was the only decision tool that resulted in a significant reduction of decisional conflict in comparison with control groups [19]. This result is atypical of the high-quality evidence from the Cochrane Review that demonstrated the ability of decision aids to reduce decisional conflict [8]. The failure of the decision tools to reduce decisional conflict in Hochlehnert et al [23] and Weymann et al [21] may be due to the presence of computerized decision aids in both control and intervention groups rather than the control group receiving usual care. Therefore, these studies effectively compare the difference between different types of computerized decision aids and their effects on patient-centered outcomes. The addition of a control group without decision aid access in the studies by Hochlehnert et al [23] and Weymann et al [21] would have allowed for the evaluation of the SDM tools’ effectiveness in comparison with usual care. Additional factors may have also affected efficacy of the computerized decision aids in reducing decisional conflict. Whereas Weymann et al [21] found no significant effects for decisional conflict, they did observe an impact on knowledge, suggesting that the tool used in the study may act more as an educational rather than a decisional tool.

EHR portals that function as a decision support system for both patients and physicians present a unique opportunity to manage care from the home. The MyAsthma portal for pediatric asthma did not have an effect on quality of life measures but did result in decreased days of work missed by parents of pediatric patients and a reduction of asthma flares [22]. This suggests that EHR portals can help patients or family members self-manage chronic illnesses. The use of EHR portals to facilitate SDM is fitting as electronic medical record utilization is considerable; approximately 75% of the Canadian and US physicians use electronic medical records [25,26].

The information presented in a decision tool is of importance to achieving meaningful patient-centered outcomes. Hochlehnert et al [23] demonstrated that satisfaction of tool information, tool usefulness, and tool introduction was significantly associated with satisfaction of treatment decision and decreased decisional conflict for fibromyalgia patients. Meropol et al [19] also reported an increase in patient satisfaction in the intervention group and also found this to be related to patients’ education level and baseline quality of life scores. Patients with higher levels of education and poorer physical functioning were found to be more satisfied following tool use. Conversely, while van der Krieke et al [20] did not find any overall difference in patient satisfaction, it was found that those in the intervention group who had received the opportunity to use the tool reported lower satisfaction compared with those who did not. This finding may have been a result of poor implementation of the study protocol or may have been due to other factors such as the format of the computerized decision aid use, the setting in which the tool was used, and whether guidance on tool use is provided. It is, therefore, important to consider contextual factors that may influence the use and effectiveness of computerized decision aids. Evidence-based frameworks, such as the Ottawa Decision Support Framework, have been used to develop and evaluate patient decision aids [27]. Further research should focus on determining which formats or, more specifically, which features of computerized decision aids are most helpful for patients.

It is possible that patients may benefit from decision tools without the need for specialized communications training or extra involvement of physicians. The computerized information tool for fibromyalgia patients was tested with and without consultation of a physician specially trained in facilitating SDM. There were no statistical differences between groups on any patient-centered outcomes, including decisional conflict or satisfaction with decision [23]. Additionally, the intervention arms for the CONNECT Web-based communication aid with and without a summary report for the patient’s physician did not differ in any patient-centered outcomes [19]. Time constraints are often cited as a barrier to the implementation of SDM [28]; therefore, reduced physician involvement may lead to greater acceptance of SDM tools.

A computerized decision aid, such as Adjuvant!, can present the risks and benefits of treatments to the patients and allow them to consider their preferences and values when making treatment decisions. This may result in a reduction in therapies that are not in line with patient preferences or disease severity and, consequently, can reduce treatment cost: Adjuvant! demonstrated a reduction in adjuvant therapy, such as chemotherapy, in breast cancer patients and was effective at decreasing adjuvant treatment in patients with low tumor severity [24].

Only one study compared computerized decision aids against a nontechnological decision aid. Adjuvant! resulted in decreased use of adjuvant therapy in comparison with control group participants who received informational pamphlets about adjuvant therapy [24]. This suggests that the computerized aid was more effective than a traditional pamphlet in communicating information on treatment options and expectations. Further research that compares traditional noncomputerized decision aids against computerized decision aids in serious illness would be useful; computerized decision aids may be more sophisticated in their ability to communicate health information to patients than traditional aids because of their greater degree of interactivity and personalization.

The tools discussed in this review are relatively simple from a technological perspective. There is potential for greater detail and personalization in SDM with the advent of more advanced decision support tools and the widespread of EHRs. For example, it has been suggested that dynamic clinical data mining can be used to provide real-time decision support. Search engine queries of a population database built on deidentified EHR would provide clinical data support using prior clinical cases, relevant statistics, scholarly resources, and protocols [29]. However, to properly facilitate SDM, a patient interface would need to be included. Additionally, integration of genomic data into EHRs can provide genomic risk scores and personalized risk information to the patient and help guide SDM [30]. Finally, a move toward more universal decision support with the ability to update based on new research findings, patient experience, and postdecision outcomes may be more cost-effective than separate and static decision aids for each disease and treatment options.

### Limitations

A limitation of this study was the quality of the RCTs selected for review. The RCTs included in this review were either at High Risk of Bias or Unclear Risk of Bias. Risk of bias should be considered when assessing the strength of evidence provided by the RCTs in this review. The literature search was also only limited to published papers and is therefore subject to publication bias. Furthermore, the search was only limited to PubMed and Scopus databases. Although these databases consist of an extensive amount of literature on the topic, the results may not have been representative of the entirety of the literature. Additionally, secondary search strategies were not performed. The study was also not registered in PROSPERO, which limits the study in terms of adhering to current best practices for systematic reviews. Finally, although quality assessment of the selected papers was completed by 2 investigators, the study is limited because of the fact that the selection of papers was completed by only a single investigator.

### Conclusions

Most computerized decision aids improved at least one patient-centered outcome. The RCTs differed in patient outcomes measured and the efficacy of decision aids in improving the aspects of SDM. All RCTs identified were at High Risk of Bias or Unclear Risk of Bias according to a modified version of the Cochrane Risk of Bias Tool for Quality Assessment of Randomized Controlled Trials. Efforts should be made to improve the quality of RCTs testing SDM aids in serious illness.

## Abbreviations

CLBP: chronic lower back pain
CST: communication skills training
EHR: electronic health record
IHCAs: interaction health communication applications
PRISMA: preferred reporting items for systematic reviews and meta-analyses
PROSPERO: International Prospective Register of Systematic Reviews
RCTs: randomized controlled trials
SDM: shared decision making
T2D: type 2 diabetes

## Appendix

Multimedia Appendix 1 Study description, effect of decision tools on patient-centered outcomes, and risk of bias.
