# Supplementary material for: Computerized Decision Aids for Shared Decision Making in Serious Illness: Systematic Review
Source: JMIR Med Inform. 2017 Oct 6;5(4):e36. doi: 10.2196/medinform.6405 (PMC5650682; doi:10.2196/medinform.6405)
Supplement: Multimedia Appendix 1 [file medinform_v5i4e36_app1.pdf]

Source	Study population	Computerized decision aid	Intervention	Control	Effect on patient-centered outcomes	Study limitations	Risk of bias
Meropol et al, 2013 [19]	*Setting*: Three academic medical centers.<br><br>*Recruitment*: Review of patient schedules and medical information before first consultation.<br><br>*Inclusion criteria*: First outpatient consultation with a medical oncologist; solid metastatic tumor; ≥18 years; able to read English.<br><br>*Exclusion criteria*: Not applicable (N/A).<br><br>*Baseline sample*: A total of 743 cancer patients, randomized to three study arms (two intervention, one	*Tool format*: Web-based tool.<br><br>The CONNECT tool consisted of a survey and communications training. The survey assessed patient preference and lifestyle characteristics.<br><br>The communication training provided guidance on how to communicate with physicians.	*Intervention 1*: CONNECT Web-based communication aid.<br><br>*Intervention 2*: CONNECT Web-based communication aid with physician summary report of patient survey results.	Usual care and direction to the National Cancer Institute's website.	No difference between Interventions 1 and 2 on any satisfaction or decisional conflict responses (*P*>.05); therefore, intervention groups were merged.<br><br>*Merged groups*: Reduced decisional conflict (*P*<.01), increased satisfaction with treatment decision (*P*<.001), higher levels of satisfaction with physician discussion on format of communication and quality of life (*P*<.05).<br><br>No difference in satisfaction with physician discussion on diagnosis/prognosis, treatment options, or community services (*P*>.05).<br>Decreased	Homogenous sample predominantly drawn from large cancer centers.<br><br>Many eligible patients declined participation.<br><br>Access was limited to those with personal Internet access or those able to arrive early for their appointment.<br><br>Control group patients' access to the National Cancer Institute's website may have reduced differences between study arms.	*High risk of bias.*<br><br>Selection bias due to inadequate generation of random groups.

	control)<br><br>*Final sample*: A total of 629 patients completed post consultation or 3-month follow-up surveys				expectations of severe side effects with therapy (*P*<.05).<br><br>Those in intervention groups found decisions easier to reach by taking part in the program (*P*<.01) and were more satisfied with their treatment decisions (*P*<.001).<br><br>Patients with lower baseline quality of life (QOL) in the intervention groups had greater satisfaction with communication about prognosis/diagnosis (*P*<.05), QOL (interaction *P*<.05), format of communication (interaction *P*<.05), and overall communication (interaction *P*<.05).		
van der Krieke et al, 2013	*Setting*: Mental health institution.	*Tool format*: Web-based tool.	Care as described in the local	Care as described in the local	No difference in perceived involvement in	Low response rate and moderate	*Unclear risk of bias.*

[20]	*Recruitment*: Two outpatient teams for psychosis (early intervention for patients with a first episode of psychosis and rehabilitation for chronic schizophrenia).<br><br>*Inclusion criteria*: Nonaffective psychosis; 21-65 years; fluent in Dutch.<br><br>*Exclusion criteria*: N/A.<br>*Baseline sample*: A total of 250 Dutch patients randomized into an intervention group (n=124) and a control group (n=73).<br><br>*Final sample*: A total of 73 patients completed follow-up measurements:	Web-based decision tool that consisted of a questionnaire on patient care needs and a catalogue of treatment options based on questionnaire answers.	disease management program for psychosis and access to the Web-based decision tool, and descriptions of the treatment, information on clinicians involved in the treatments, and anecdotal patient experiences of treatment options.	disease management program for the treatment of psychosis.	medical decision making (*P*>.05).<br><br>No difference in self-reported satisfaction (*P*>.05).<br><br>Patients in the intervention group (received intervention vs did not) differed in self-reported satisfaction with care (*P*=.02); patients who received the intervention were less satisfied than patients who did not.	participation rate.<br><br>Decision aid was not offered to all patients in the intervention group.<br><br>Treatment evaluation meetings (at which the shared decision making [SDM] process was to occur) did not always take place.<br><br>Lack of uniform framing of the Web-based intervention, possibly influencing patients' expectations and evaluation of the tool.	Insufficient information regarding blinding process.

	40 in the intervention group and 33 in the control group. Of the 40 patients in the intervention group, 30 received the allocated intervention whereas 10 did not.							
Weymann et al, 2015 [21]	*Setting*: No central setting, Web-based trial.<br><br>*Recruitment*: Website and flyers of pension funds, insurance companies, and outpatient treatment networks; mailing list of a Web-based panel at a university.<br><br>*Inclusion criteria*:<br>≥18 years; access to the Internet and computer/Interne	*Tool format*: Web-based tool.<br><br>The tool provided information on diabetes or low back pain and treatment options in a dialogue format tailored to patient characteristics such as knowledge and information preferences.	Access to the Web-based tailored communication tool.	Static, nondialogue format, Web-based communication tool.	*Intention-to-treat analysis (n=551)*: No significant difference was found in knowledge following first visit in tailored versus control group (*P*=.53); however, there was a significant difference between users with T2D and CLBP (*P*<.001), with higher knowledge scores in the T2D group.<br><br>No difference in decisional conflict and preparation for decision making	Only patients with Internet access included.<br><br>Diagnosis was self-assessed.<br><br>Outcome criteria not measured at baseline.<br><br>Did not assess potential confounders.<br><br>Use of dialogue-based system may have allowed	*High risk of bias.*<br><br>Detection bias due to indirect knowledge of intervention.

	t literacy; self-reported diagnosis of type 2 diabetes (T2D) or chronic lower back pain (CLBP).<br><br>*Exclusion criteria*:<br>N/A.<br><br>*Baseline sample*:<br>A total of 551 participants with analyzable data, that is, basic demographic information (n=173 T2D and n=378 CLBP) randomly assigned to the tailored condition (n=283) or control condition (n=278).<br><br>*Final sample*:<br>A total of 360 participants at postintervention assessment (ie, after first visit); 295 participants				($P>.05$); however, decisional conflict was lower in the CLBP group than in the T2D group ($P<.001$) after the first visit.<br><br>*Sensitivity/available case analysis*:<br><br>Increased knowledge after first visit in the intervention group versus control group ($P<.05$).<br><br>Increased emotional well-being in the intervention group at the 3-month follow-up ($P<.05$).	blinded participants to identify the intervention.	

	with at least one of three outcomes at the 3-month follow-up (n=93 T2D and n=202 CLBP) with 146 participants in the tailored condition and 149 in the control condition.						
Fiks et al, 2015 [22]	*Setting*:<br>One urban and two suburban primary care practices.<br><br>*Recruitment*:<br>Rosters generated from an electronic health record (EHR); referrals from clinicians and coordinators.<br><br>*Inclusion criteria*:<br>Parents or legal guardians of children aged 6 to 12 years with persistent	*Tool format*:<br>EHR portal.<br><br>MyAsthma EHR decision support portal for clinicians and parents that provided treatment plans and goals, symptom and side effect tracking, and educational content.	Access to MyAsthma EHR decision portal with monthly portal surveys; surveys guided guideline-based decision support for families and clinicians to engage in conversation regarding management of asthma or side effects.	Usual care with clinician-only access to decision support system.	No effect on number of school days missed (*P*=.20).<br><br>Significant effect on parental workdays missed (*P*=.001).<br><br>No effect on parental activation (*P*=.90).<br><br>No differences in quality of life (*P*>.30).<br><br>Reduced asthma flares (*P*=.02).<br><br>More frequent portal use among parents of children	Study participants were a convenience sample.<br><br>Randomization did not fully balance asthma severity between intervention and control groups.<br><br>Results may not generalize to self-management tools for asthma.	*High risk of bias.*<br><br>Selection bias due to inadequate randomization.

	asthma; receiving care at study site.<br>*Exclusion criteria*:<br>Parents of children whose asthma was not a primary/current health concern; those not currently taking medication.<br><br>*Baseline sample*:<br>A total of 60 families, 30 families per arm (intervention and control).<br><br>*Final sample*:<br>A total of 53 families, 87% (26/30) in the intervention group and 90% (27/30) in the control group; completed 6-month outcome measures.				with moderate or severe asthma.<br><br>No significant difference between study arms in satisfaction with asthma care.<br>Compared with the control group, families of intervention group reported fewer emergency department visits (3 vs 9) and hospitalization over 6 months (0 vs 2).	Outcomes primarily based on parent report.	
Hochlehnert et al, 2006 [23]	*Setting*:<br>Academic center.	*Tool format*:<br>interactive computer	Computerized information tool and	Computerized information tool and	No statistical significance was found in satisfaction	Insufficient information regarding the	*Unclear risk of bias.*

	Recruitment: N/A.<br>*Inclusion criteria*: Fibromyalgia patients.<br><br>*Exclusion criteria*: N/A.<br><br>*Baseline and final sample*: A total of 75 patients with fibromyalgia; mean age 50 years; 93% (70/75) female.	application.<br><br>The tool provided patients with information on symptoms, diagnosis, pathogenesis, treatment outcomes, and prognosis.	subsequent SDM opportunity with specially trained clinicians with opportunities for additional questions and feedback.	standard doctor and without subsequent SDM opportunity, with no additional opportunity for feedback or additional questions.	with decision, and decision conflict was found between groups (P>.05).<br><br>No statistically significant differences found between groups in assessment of information tool (P>.05); therefore, both groups were merged together.<br><br>*Merged groups*: Positive correlation between assessment of the information and satisfaction with the decision (r=.42, P<.01).<br><br>Assessment of the information was associated with decreased decisional conflict (r=−.41, P<.01).<br><br>Perceived tool usefulness was positively associated with decision satisfaction (r=.29, P<.05).	blinding process.	Insufficient information regarding the blinding process.

					Satisfaction with tool introduction (training) was positively associated with decision satisfaction (*r*=.3, *P*<.05).		
Peele et al, 2005 [24]	*Setting*:<br>Five academic oncology practices and nine community-based practices.<br><br>*Recruitment*:<br>N/A.<br><br>*Inclusion criteria*:<br>Breast cancer diagnosis; completed primary surgical treatment; candidates for adjuvant therapy; no prior history of breast cancer.<br>*Exclusion criteria*:<br>N/A.<br><br>*Baseline sample*:<br>A total of 432 patients were	*Tool format*:<br>Interactive computer application. Adjuvant! produced prognostic estimates of survival with and without adjuvant therapy and estimates of the efficacy of adjuvant therapy options.	Access to Adjuvant! decision aid.	Informational pamphlet on adjuvant therapy.	Decreased use of adjuvant therapy in intervention group (*P*=.039).<br><br>Decreased adjuvant therapy in intervention group patients with low tumor severity (58%, 35/60, *P*=.003), whereas intervention patients with high tumor severity were significantly more likely to choose adjuvant therapy (99%, 68/69, *P*=.04).<br><br>Women were more likely to choose adjuvant therapy if they had higher tumor severity, were younger, and had a university-based	Neither patients nor clinicians were blinded.<br><br>Higher proportion of university-based physicians in the intervention group.	*High risk of bias.*<br><br>Performance and detection bias since neither patients nor clinicians were blinded.<br><br>Selection bias due to disproportionat e randomization of university-based physicians.

	randomized to the intervention arm (n=250) and control arm (n=182).<br><br>*Final sample*: A total of 386 patients with complete data; 226 patients in the intervention arm and 160 patients in the control arm; mean age 62 years.				physician (all *P*<.01).		
